# Safety, tolerability, and preliminary efficacy of nadunolimab, an anti-IL- 1 receptor accessory protein monoclonal antibody, in combination with pembrolizumab in patients with solid tumors

**DOI:** 10.1007/s10637-025-01538-3

**Published:** 2025-05-01

**Authors:** Roger B. Cohen, Antonio Jimeno, Jennifer Hreno, Lova Sun, Marie Wallén-Öhman, Camilla Rydberg Millrud, Annika Sanfridson, Ignacio Garcia-Ribas

**Affiliations:** 1 Division of Hematology-Oncology, University of Pennsylvania, Philadelphia, PA USA; 2 Department of Medicine, Division of Medical Oncology, Developmental Therapeutics Program, University of Colorado School of Medicine, Aurora, CO USA; 3 Present Address: Cantargia AB, Lund, Sweden

**Keywords:** Nadunolimab, Solid tumors, Efficacy, Safety, Pharmacokinetics, Biomarkers

## Abstract

Interleukin (IL)-1 signaling has an essential role in tumor progression and immunosuppression and is linked to acquired resistance to anti-PD-1/PD-L1 treatment. Nadunolimab is an IL1RAP (IL-1 receptor accessory protein)-targeting antibody that blocks IL-1α/IL-1β signaling and has enhanced antibody-dependent cellular cytotoxicity. We investigated the safety and preliminary efficacy of nadunolimab with pembrolizumab in patients with metastatic solid tumors who had progressed on previous checkpoint inhibitor treatment, suggesting acquired checkpoint inhibitor resistance (NCT04452214). This phase 1b trial enrolled patients with metastatic disease who had exhausted or declined standard-of-care alternatives. Patients received nadunolimab (5 mg/kg) and standard-dose pembrolizumab. The primary objective was to assess safety. Secondary objectives were anti-tumor response as per iRECIST, pharmacokinetics, and changes in immune mediators. Fifteen patients with stage IV cancer (head and neck squamous cell carcinoma, non-small cell lung cancer, melanoma) entered the trial. Grade ≥ 3 adverse events were reported for 7 patients (47%). There was one dose-limiting toxicity of febrile neutropenia. The most frequent grade ≥ 3 adverse event was dysphagia (two patients). Seven patients (47%) had reductions in target lesion size. Median iPFS was 3.4 months (95% CI 1.4–8.6). Median OS was 19.7 months (95% CI 4.3–28.7) with 67% 1-year survival. Survival was significantly longer in patients with higher baseline tumor infiltration of CD163 + macrophages and natural killer cells and in patients with reduced on-treatment circulating IL-6 levels or neutrophil-to-lymphocyte ratio. Nadunolimab with pembrolizumab had an acceptable safety profile, and prolonged disease control was observed in a subset of patients. The results support further development of nadunolimab in combination with checkpoint inhibitors.

**Supplementary Information:**

The online version contains supplementary material available at 10.1007/s10637-025-01538-3.

## Introduction

Monoclonal antibody inhibitors of the programmed death (ligand) 1 (PD- 1/PD-L1) axis play a prominent role in the treatment of many solid tumors and act by reversing the blockage of T cell function, such that tumor-reactive T cells can proceed with tumor cell suppression and killing [1]. Unfortunately, a substantial proportion of patients do not respond to treatment, and most responders eventually develop treatment resistance [2]. There is accumulating evidence of crosstalk between PD- 1/PD-L1 and interleukin (IL)− 1 pathways within the tumor microenvironment (TME), leading to treatment resistance to immune checkpoint inhibitors (ICIs) due to immunosuppression [3–6].

The IL- 1 signaling system is an important component of chronic inflammation, tumor progression, metastasis, immune evasion, immunosuppression, and treatment resistance [7–10]. The IL- 1 ligands, IL- 1α and IL- 1β, bind to the IL- 1 receptor, leading to an interaction with the IL- 1 receptor accessory protein (IL1RAP), which is essential for IL- 1 mediated signal transduction [11]. IL1RAP plays a key role in the development of cancer, being highly expressed by tumor cells and myeloid cells, and it is involved in the generation, expansion, immunosuppressive function, and recruitment of myeloid cells, such as myeloid-derived suppressor cells and M2 macrophages, into the TME [12–18]. Synergistic effects of treatment with IL- 1 family blockade and immunotherapies on tumor growth inhibition have been observed in mouse models [6, 9, 19]. A combination of IL1RAP pathway suppression and ICI treatment may promote a TME with improved immune cell functionality, potentially enhancing or restoring an effective anti-tumor response.

Nadunolimab is a fully humanized, monoclonal, antibody-dependent cell-mediated cytotoxicity (ADCC)-enhanced IgG1 antibody that targets IL1RAP. Nadunolimab inhibits IL- 1α and IL- 1β signaling and has shown strong anti-tumor effects in the presence of platinum and non-platinum-based chemotherapy in preclinical in vitro and in vivo studies [20, 21]. In addition, nadunolimab also mediates tumor cell killing via Fcγ-receptor interactions with effector cells, for instance, NK cells and macrophages, to effect ADCC and antibody-dependent cellular phagocytosis [22–25].

A phase 1 study in heavily pre-treated patients with advanced, treatment-refractory solid tumors found that nadunolimab monotherapy was well tolerated in doses up to 10 mg/kg. There were no partial or complete responses per RECIST1.1. Grade ≥ 3 treatment-emergent adverse events (TEAEs) were reported for 10 patients [16]. In a phase 2a dose escalation and expansion study, nadunolimab showed promising efficacy when combined with standard-of-care chemotherapy in patients with untreated locally advanced/metastatic pancreatic cancer or metastatic non-small cell lung cancer (NSCLC) [26–28].

Based on emerging evidence indicating the role of IL1RAP in resistance to PD- 1/PD-L1 inhibitors, we investigated whether the combination of nadunolimab and pembrolizumab was safe and tolerable and whether the addition of nadunolimab could overcome acquired resistance to ICIs. The current study, CIRIFOUR (NCT04452214), was a phase 1b, open-label trial of nadunolimab in combination with pembrolizumab. The primary objective was to explore safety and to additionally describe preliminary efficacy of the combination, including biomarker analysis.

## Methods

### Study design and patients

Eligible patients were aged ≥ 18 years with metastatic NSCLC, head and neck squamous cell carcinoma (HNSCC), or malignant melanoma, who had progressed after at least 12 weeks of treatment with an ICI targeting the PD- 1/PD-L1 pathway (alone or in combination with chemotherapy) after initially achieving stable disease (SD) or better. Eligible patients had exhausted or declined available standard therapy and had measurable disease by iRECIST and an Eastern Cooperative Oncology Group performance score ≤ 1. Eligibility criteria are listed in the supplement.

The study enrolled 15 patients to assess the safety of 5 mg/kg of nadunolimab in combination with a standard dose and schedule of pembrolizumab, with an option to de-escalate nadunolimab in case of treatment-emergent toxicity. Dose selection and additional study design features are provided in the supplement.

### Ethics approval

This study was conducted in accordance with Good Clinical Practice guidelines, the Declaration of Helsinki, and applicable ethical and regulatory requirements. The study protocol was approved by independent review boards at each participating site.

Safety data were periodically reviewed by a Safety Review Committee comprised of study investigators and a medical representative of the sponsor.

### Treatment

Patients were treated with nadunolimab at 5 mg/kg weekly and pembrolizumab 200 mg every 3 weeks in cycles of 21 days. A reduced (priming) dose (0.5 mg/kg) of nadunolimab was given on cycle 1, day -7, to lower the risk of infusion-related reactions [16]. Full nadunolimab doses (5 mg/kg) commenced on cycle 1, day 1, and were administered on days 1, 8, and 15; every 21 days in cycles 1 and 2; and on days 1 and 8 thereafter. Pembrolizumab was administered on day 1 of each cycle, after the nadunolimab infusion.

The day 8 nadunolimab dose was omitted from cycle 9 onward for four patients with documented clinical benefit (SD or better by iRECIST) after eight cycles, at the discretion of the investigator and in accordance with the protocol. Patients that discontinued pembrolizumab could remain on nadunolimab monotherapy at the discretion of the investigator. Treatment with nadunolimab could continue until disease progression or for up to 1 year. Patients with documented clinical benefit were allowed to continue receiving study treatment beyond 1 year with sponsor approval.

### Objectives and endpoints

The primary study objective was the assessment of safety based on TEAEs, dose-limiting toxicities (DLTs), physical examination findings, vital sign measurements, electrocardiograph recordings, and standard clinical laboratory measurements.

Secondary objectives included preliminary signs of clinical efficacy based on iRECIST, changes in serum IL- 6 and C-reactive protein with treatment, and assessment of serum drug concentrations of nadunolimab and pembrolizumab when given in combination [29]. Changes in disease-related inflammatory biomarkers in serum and tumor tissue were exploratory objectives.

Clinical response to therapy was assessed in terms of the overall response rate (ORR), immune progression-free survival (iPFS), overall survival (OS), duration of response, and time to response. The immune disease control rate (iDCR) was the best response of immune complete response, immune partial response (iPR), or immune stable disease for at least 6 weeks.

### Translational analysis

#### Immunohistochemistry analysis

Expression of IL1RAP (polyclonal rbIgG; Cantargia) and PD-L1 (AB_2819099) by tumor, stromal, and immune cells was assessed in screening core needle biopsies (12 patients) and on-treatment biopsies after 4 weeks of treatment (8 patients) using immunohistochemistry. Screening and on-treatment biopsies could be from different tumors/sites. Biopsies were formalin-fixed and paraffin-embedded. The expression of IL1RAP was quantified by H-score ([1 × % weakly-stained cells] + [2 × % moderately-stained cells] + [3 × % strongly-stained cells]) on tumor cells [30]. IL1RAP expression levels were quantified in stroma as none, low, medium, or high and as semi-quantitative percentages on infiltrating immune cells. PD-L1 expression was measured as the percentage of positive tumor cells and the semi-quantitative percentage of PD-L1 + immune cells.

Biopsies were stained for CD8 (AB_929437), NK cell marker NKp46 (AB_3096083), and CD163 (M2 anti-inflammatory macrophages, AB_2074540). Percentages of NKp46 + and CD8 + immune cells in the tumor nests were estimated, and the levels in the stroma were scored as none, low, medium, or high. CD163 + immune cells were scored as none, low, medium, or high within the biopsy. The staining was evaluated and scored by the same pathologist blinded to patient status and indication.

#### Serum biomarkers

Patient serum samples were collected at screening and repeatedly during treatment. Serum levels of cytokines were evaluated using the Olink Target 96 Immuno-Oncology panel (Olink, Uppsala, Sweden). IL- 6 (LLOQ 0.633 pg/mL) was analyzed with a validated V-plex Meso Scale Discovery (MSD, Maryland, USA). Hematology parameters and C-reactive protein levels were analyzed at local hospital laboratories at the investigator sites.

### Statistical analysis

The data cutoff date at study end was August 24, 2023. Safety and efficacy were assessed on the total patient population. Efficacy was also assessed in subgroups determined by survival period either greater than or equal to, or less than, the median observed survival. Tumor response was determined by iRECIST. Overall survival was assessed from the date of the priming dose of nadunolimab until study end. Time-to-event estimates used Kaplan–Meier methods. Biomarker variables were summarized using descriptive statistics overall and in patients grouped by observed median OS. Baseline biomarker levels were compared using the Mann–Whitney test, and over time using REML with Sidak multiple correction. Post hoc comparisons of baseline and on-treatment cytokine levels were performed using the multiple Wilcoxon test, and cytokine levels in treated blood using Friedman test with Dunn’s multiple comparison test.

## Results

### Patients

Fifteen patients were enrolled at three sites in the USA between September 2020 and July 2021. All patients had stage IV disease at study entry. Nine patients had HNSCC, five had NSCLC, and one had melanoma (Supplemental Table 1). Patterns of prior treatment and the duration of prior treatment with ICIs were heterogeneous. All patients had received prior treatment with ICIs for at least 3 months and for up to 30 months, with SD or better as the best response before progression (Supplemental Fig. 1).

The median duration of follow-up was 17 months (range 2–29). All patients received at least two cycles of nadunolimab and pembrolizumab. The median number of doses was 11 (range 5–48) for nadunolimab and 5 (2–37) for pembrolizumab, with a median treatment duration of 118 days (range 47–826) and 111 days (range 34–819), respectively. The median relative dose intensity was 93% for both nadunolimab and pembrolizumab.

At the data cut-off date, 13 patients had discontinued nadunolimab and pembrolizumab treatment due to PD. One patient continued treatment beyond study completion under a single patient IND, while one was transferred to other therapies by the investigator. Nine patients had at least one systemic antineoplastic treatment after nadunolimab discontinuation.

### Safety

Nadunolimab with pembrolizumab was well tolerated. The most frequently reported TEAEs were fatigue (*n* = 8), pruritus (*n* = 6), and hypotension, arthralgia, dyspnea, and diarrhea (*n* = 4 each) (Supplemental Table 2). No infusion-related reactions were reported. Grade ≥ 3 TEAEs were reported by seven patients (Supplemental Table 3). Grade ≥ 3 TEAEs in two patients were considered by the investigator to be treatment-related: grade 3 pneumonitis in one patient and grade 3 febrile neutropenia in the other. The latter event was considered a DLT per protocol.

One patient died due to *Pneumocystis jirovecii* infection on day 108, which was considered by the investigator to be unrelated to treatment.

Six patients reported 13 treatment-emergent serious adverse events (Supplemental Table 4). Other than the event of DLT, all were assessed by the investigator as unrelated to treatment.

One patient discontinued pembrolizumab treatment due to a TEAE (pneumonitis). Six patients had a treatment interruption. Two patients had the dose of nadunolimab reduced to 2.5 mg/kg due to either febrile neutropenia or pneumonitis. There were no important laboratory or vital signs changes during the study.

### Efficacy analysis

**Fig. 1:**
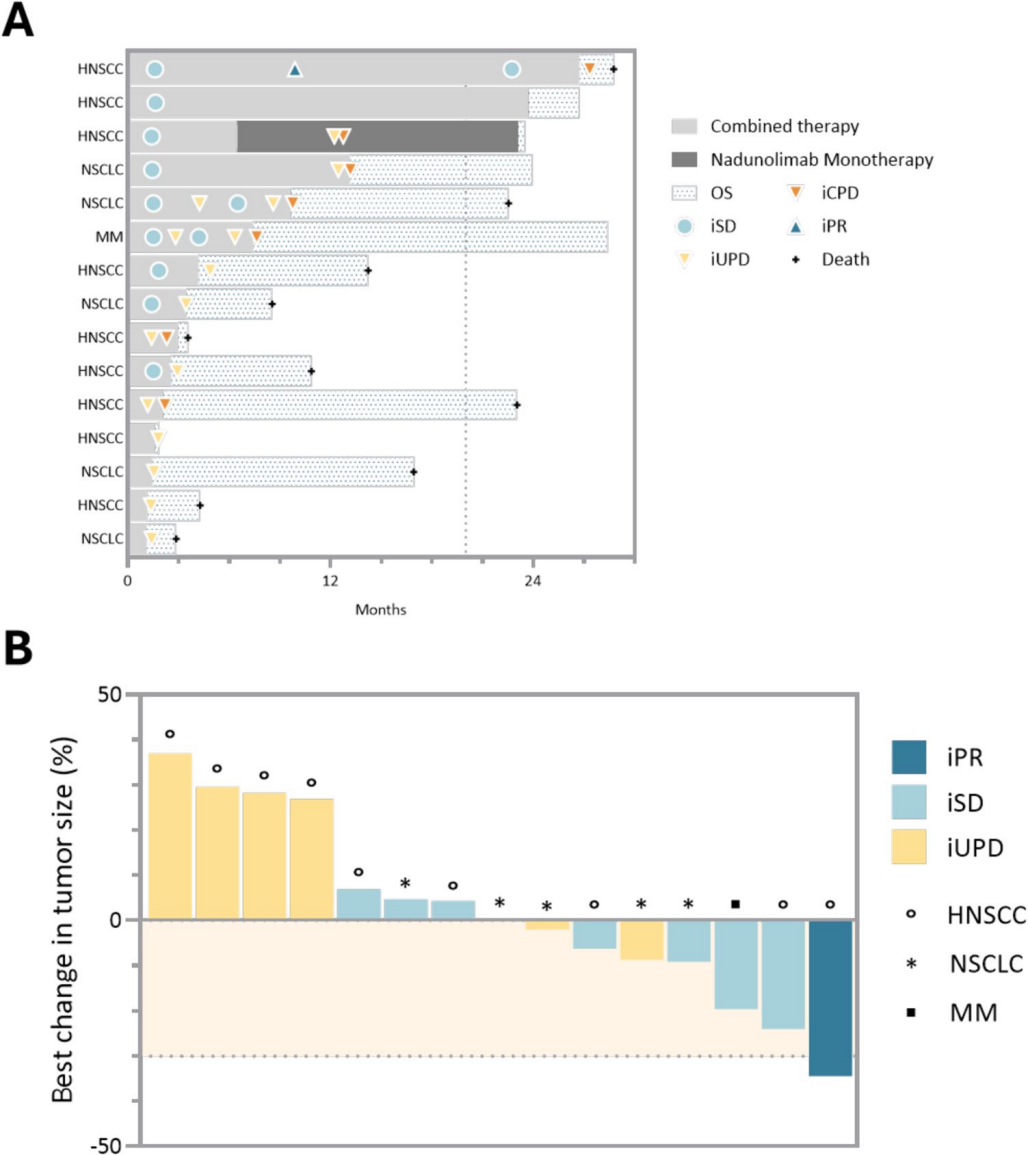
Clinical response (iRECIST) (safety population). **A** Swimmer plot of tumor response (iRECIST) and **B** waterfall plot of maximum percentage change in sum of diameters of target lesions (mm) from baseline. Patients with clinical benefit were allowed to continue on nadunolimab monotherapy beyond progression. One patient with HNSCC (7%) had confirmed iPR with a duration of response of 17.4 months. Eight patients (53%) had iSD as the best response. HNSCC, head and neck squamous cell carcinoma; NSCLC, non-small cell lung cancer; MM, malignant melanoma. OS, overall survival; iCPD, immune confirmed progressive disease; iPR, immune partial response; iSD, immune stable disease (present for more than 6 weeks); iUPD, immune unconfirmed progressive disease

The overall response rate was 7% based on one patient with HNSCC who had a best response of iPR and a duration of response of 17.4 months (Fig. 1A). This patient achieved PR after 15 cycles. The iDCR was 60% (95% CI 32–84) (Table 1). Seven patients (47%) had reductions in target lesion size (Fig. 1B). Median iPFS was 3.4 months (95% CI 1.4–8.6), and the iPFS rate at 6 months was 40%, 27% at 12 months, and 13% at 24 months. Five patients were alive at the end of the study. Median OS was 19.7 months (95% CI 4.3–28.7) with a survival probability of 67% at 12 months and 40% at 24 months (Table 1). There was no evidence for a differential effect of nadunolimab with pembrolizumab on different tumor types (Supplemental Fig. 2) and no clear relationship between prior treatment type or duration and OS in the current study. However, patients with longer OS had a lower tumor burden at baseline (Supplemental Table 4).

**Table 1:** Efficacy outcomes by iRECIST (safety population)

Outcome	Total *N* = 15
Overall survival	
Median—months (95% CI)	19.7 (4.3–28.7)
Survival rate	
12 months, % (95% CI)	67 (38–88)
24 months, % (95% CI)	40 (16–68)
Response (iRECIST)	
Overall response rate % (95% CI)*	7 (0–32)
Partial response, *n* (%)	1 (7)
Stable disease, *n* (%)	8 (53)
Unconfirmed progressive disease	4 (27)
Confirmed progressive disease, *n* (%)	2 (13)
Disease control rate % (95% CI)**	60 (32–84)
Duration of response median (months)	17.4
Immune progression-free survival	
Median—months (95% CI) [	3.4 (1.4–8.6)
Survival rate	
6 months, % (95% CI)	40 (16–68)
12 months, % (95% CI)	27 (8–55)
24 months (95% CI)	13 (2–40)

Abbreviations: *CI*, confidence interval

^*^Overall response rate = complete response + partial response

^**^Disease control rate = complete response + partial response + stable disease for at least 6 weeks

Two-sided 95% exact Clopper-Pearson confidence interval

### Exposure analysis

Samples were analyzed for nadunolimab and pembrolizumab serum concentrations throughout the treatment period (Supplemental Fig. 3). Trough levels of nadunolimab showed initial accumulation and then reduced with longer dosing intervals. Pembrolizumab showed slow accumulation during the first five cycles.

### Translational analysis

#### Target expression of IL1RAP and PD-L1 on tumor biopsies

**Fig. 2:**
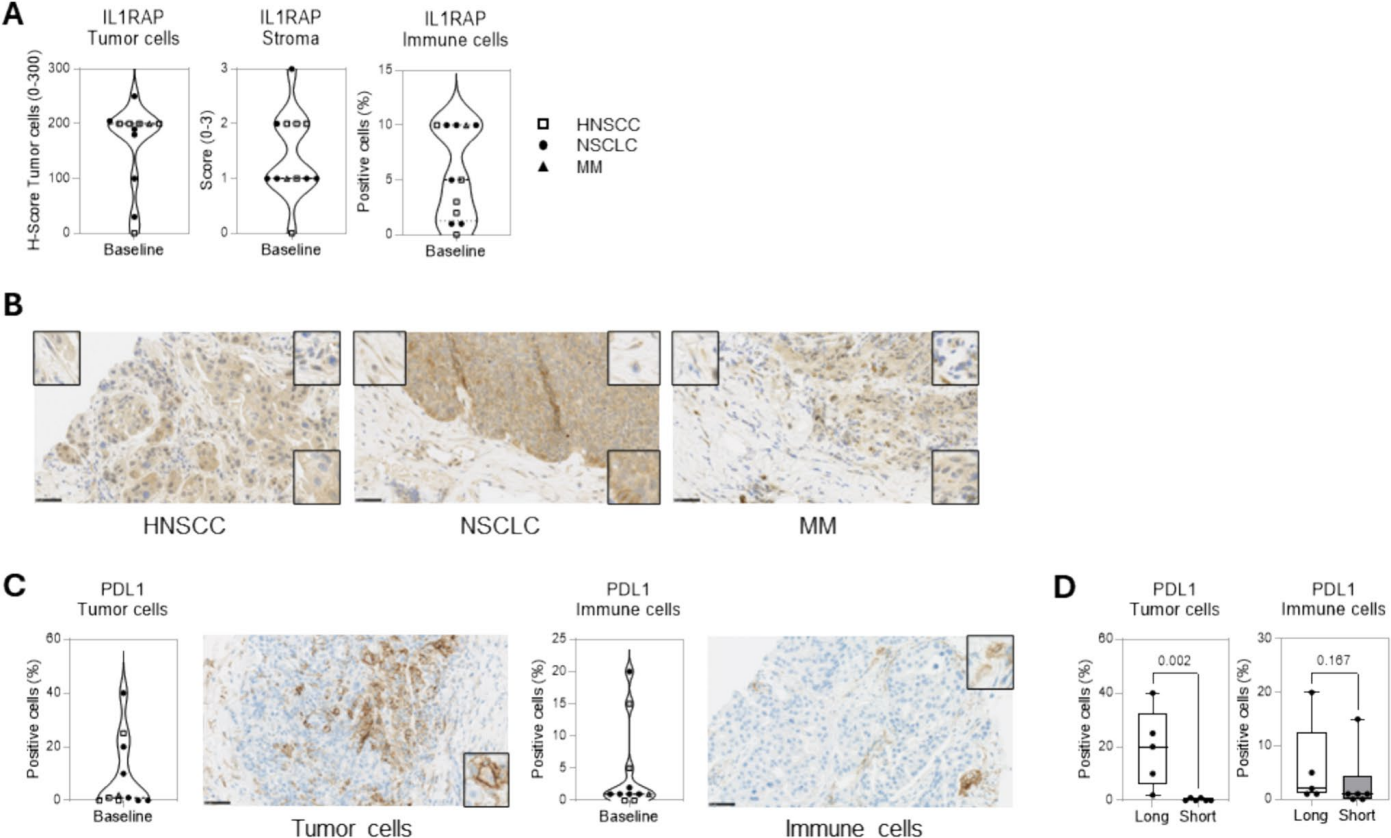
Target expression of IL1RAP and PD-L1 in tumor biopsies. Patient biopsies were collected at screening (*n* = 12) and on-treatment (cycle 2 day 8; *n* = 8) and stained for IL1RAP and PD-L1 by immunohistochemistry. **A** IL1RAP expression at baseline was evaluated on tumor cells, stroma, and immune cells. **B** Representative images are shown of IL1RAP expression in HNSCC, NSCLC, and MM. In the lower right, upper left, and upper right corners, positive IL1RAP staining of tumor cells, stroma cells, and immune cells are presented, respectively. **C** PD-L1 expression at baseline was evaluated on tumor cells and immune cells. Representative images of tumor cells and immune cells are shown. **D** Baseline PD-L1 expression was analyzed in patients divided by time since the last ICI treatment (long, > median of 80 days; or short, < median of 80 days). HNSCC, head and neck small cell cancer; ICI, immune checkpoint inhibitor; IL1RAP, interleukin 1 receptor accessory protein; MM, malignant melanoma; NSCLC, non-small cell lung cancer; PD-L1, programmed cell death ligand 1

Twelve screening biopsies (six patients with HNSCC, five with NSCLC, one with melanoma) were used to evaluate target expression of IL1RAP and PD-L1 using immunohistochemistry. Eleven of the screening biopsies expressed IL1RAP on tumor, stromal, and immune cells, as identified morphologically (Fig. 2A, B). One patient with NSCLC showed no measurable IL1RAP levels. PD-L1 expression was measured in 11 of the screening biopsies; one NSCLC tumor sample was exhausted and could not be analyzed. PD-L1 was expressed on tumor cells in seven biopsies and on immune cells in nine biopsies. Two biopsies showed no PD-L1 expression (Fig. 2C). Because therapeutic effects of ICIs require intact PD- 1/PD-L1 expression on immune cells as well as cancer cells, we investigated whether the duration since the last ICI treatment impacted PD-L1 expression. When divided based on median survival time, the five patients with > 80 days (range 84–847 days) since previous ICI discontinuation had higher expression of PD-L1 on tumor cells compared to six patients with < 80 days (range 28–76 days) since the last ICI treatment (Fig. 2D), potentially indicating that PD-L1 expression may have been reduced by treatment and then recovered over time.

Eight on-treatment biopsies were collected, with seven patients (three with NSCLC, three HNSCC, one melanoma) having both screening and on-treatment biopsies. No consistent treatment-related changes in IL1RAP or PD-L1 expression were evident (Supplemental Fig. 4A&B).

#### Immune cell analysis in tumor biopsies

To assess the inflammatory status and anti-tumor capacity in the TME, the presence of anti-tumor CD8 + T cells, immunosuppressive CD163 + macrophages, and NK cells, critical for the ADCC-enhanced function of nadunolimab, were analyzed in the screening biopsies using immunohistochemistry. CD8 + T cells were detected in eight biopsies, within both the tumor nest and stroma. NK cells, characterized as NKp46 + immune cells, were sparse and only detected in the tumor nests of six biopsies and in the stroma of five biopsies. Four samples had no NK cells at all. CD163 + immune cells were more frequently seen, and only one biopsy had no measurable CD163 + immune cells. No consistent treatment-related effects were observed for any of the analyzed biomarkers in the TME (Supplemental Fig. 5).

#### Analysis of peripheral cell populations and cytokine expression

**Fig. 3:**
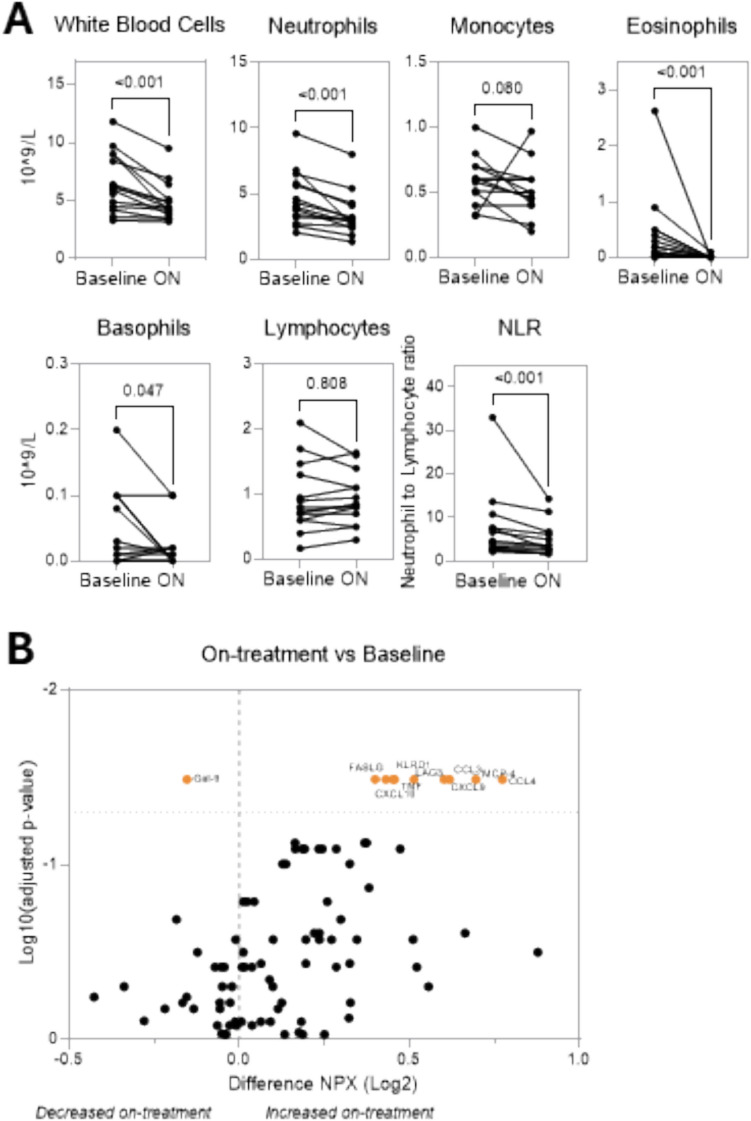
Treatment-related effects of the nadunolimab and pembrolizumab combination on peripheral cell populations and serum biomarkers. **A** Whole blood was collected before the start of treatment and on-treatment (ON; cycle 2 day 8) and analyzed for the levels of total white blood cells, neutrophils, monocytes, eosinophils, basophils, and lymphocytes (n = 15). Neutrophil-to-lymphocyte ratio (NLR) was calculated as neutrophils divided by lymphocytes. **(B)** Serum was collected as baseline and on-treatment (cycle 2 day 15) and the levels of cytokines were evaluated using the Olink Target 96 Immuno-Oncology (n = 11). Normalized protein expression (NPX) values are a relative value in Log2 scale, and differences in NPX are calculated as on-treatment value minus baseline value; a positive value increased by treatment and a negative value decreased by treatment. CCL3 and 4, C–C motif chemokine ligand 3 and 4 (macrophage inflammatory proteins- 1α and β, MIP- 1α and MIP- 1β); CXCL9 and 10, chemokine ligand 9 and 10; FASLG, Fas ligand; GAL9, galectin- 9; IL- 6, interleukin 6; KLRD1, killer cell lectin-like receptor D1; LAG3, lymphocyte activation gene 3; MCP- 4, monocyte chemoattractant protein- 4; TNF, tumor necrosis factor

Treatment-related effects on systemic inflammation were investigated by analyzing peripheral immune cell populations in whole blood at baseline and on-treatment (after 4 weeks of treatment). A significant treatment effect on peripheral immune cells was detected, with a decrease in total white blood cells, specifically neutrophils, monocytes, eosinophils, and basophils, while levels of lymphocytes were unchanged. These effects were reflected in a decreased neutrophil-to-lymphocyte ratio (NLR) (Fig. 3A).

Treatment-related effects on serum cytokine levels were analyzed using the Olink immuno-oncology panel. Increases in the T cell-related cytokines interferon-gamma (IFNγ) and chemokine ligands 9 and 10 (CXCL9 and CXCL10), involved in the migration of T cells, were detected with treatment. Other T cell and NK cell-related proteins, such as Fas ligand (FASLG), killer cell lectin-like receptor D1 (KLRD1), and lymphocyte activation gene 3 (LAG3), were also increased with treatment. The levels of the myeloid cell-secreted cytokines tumor necrosis factor (TNF), C–C motif chemokine ligand 3 and 4 (CCL3 and CCL4), and monocyte chemoattractant protein- 4 (MCP- 4) increased significantly with treatment, while Galectin- 9, involved in tumor immune evasion, decreased (Fig. 3B).

The same cytokines were increased in an in vitro experiment in which whole blood from healthy subjects was treated with nadunolimab alone (Supplemental Fig. 6), indicating that nadunolimab can directly affect the levels of these biomarkers.

#### Correlation of biomarkers with clinical benefit

**Fig. 4:**
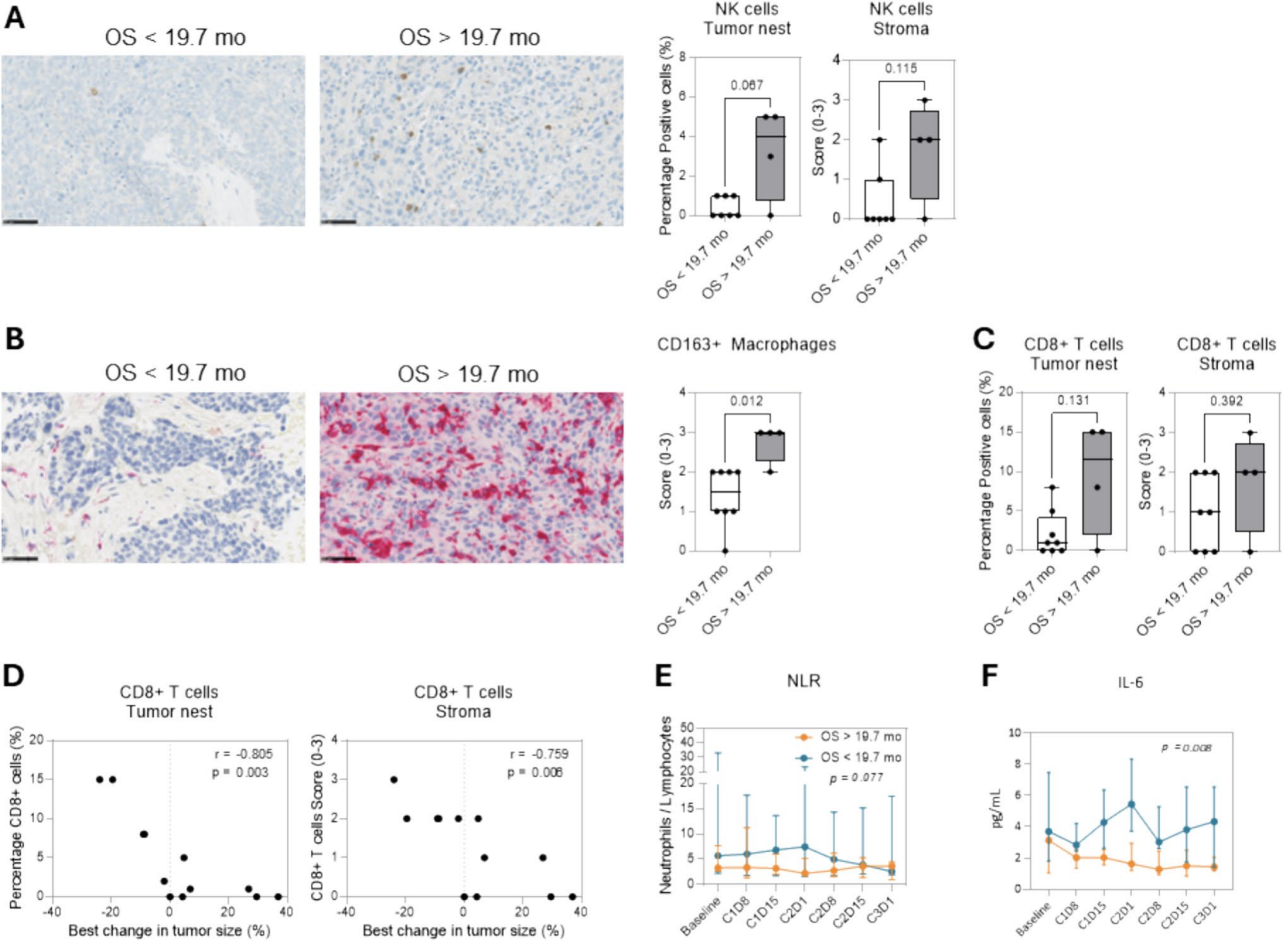
Correlations of biopsy and peripheral biomarkers with overall survival. Patients were divided on median OS (19.7 months) into those with longer OS than 19.7 months (OS > 19.7 mo) and those with shorter OS than 19.7 months (OS < 19.7 mo) and analyzed for correlation with biomarkers. **A** The percentage of NK cells, characterized as NKp46 + immune cells, in the tumor nests and stroma at baseline was determined in the two groups. Representative images of NKp46 staining are shown. **B** The level of CD163 + immune cells was scored from 0 to 3 within the whole tumor biopsy at baseline. Representative images of CD163 staining are shown. **C** Analysis of CD8 + T cells in the tumor nests and in stroma at baseline. **D** Correlation between the best change in the sum of diameters of target lesions and the levels of CD8 + T cells in the tumor nests and stroma at baseline. **E** Neutrophil-to-lymphocyte ratio at baseline and on-treatment in the two patient groups with OS above and below the median. **F** IL- 6 levels in serum at baseline and on-treatment in the two patient groups with OS above and below the median

To determine if any biomarker correlated with a clinical benefit from nadunolimab and pembrolizumab treatment, patients were divided into two groups based on the median OS of 19.7 months. The baseline characteristics were similar between the groups (Supplemental Table 4). Patients with longer OS had a trend for a higher percentage of NK cells in the tumor nests and stroma at baseline compared to those patients with a shorter OS (*p* = 0.067 and *p* = 0.115, respectively) (Fig. 4A). There was a positive correlation between CD163 + immune cells and OS, with higher levels of CD163 + immune cells detectable in patients with OS > 19.7 months (*p* = 0.012) (Fig. 4B). A trend for correlation between higher levels of CD8 + T cells in the tumor nests and longer OS (*p* = 0.131) (Fig. 4C) was also detected, and the percentage of CD8 + T cells in the tumor nests and stroma correlated inversely with change in tumor size (*p* = 0.003 and *p* = 0.006, respectively) (Fig. 4D). There was no apparent correlation between response and baseline IL1RAP or PD-L1 expression levels in tumor biopsies (Supplemental Fig. 7A, B).

Assessment of correlations between the sum of the diameter of target lesions at baseline and the different tumor biopsy markers revealed an inverse correlation between the level of NK cells in the tumor nests and the size of the target lesions (*p* = 0.042). No other correlations were observed (Supplemental Fig. 7C).

Patients with longer OS in this study had a trend for a lower NLR while on treatment (*p* = 0.077) (Fig. 4D). A significant reduction of IL- 6 levels on-treatment was observed in patients with the longest OS (*p* = 0.008) (Fig. 4E).

## Discussion

CIRIFOUR was a hypothesis-generating study with the primary objective to explore the safety of nadunolimab in combination with pembrolizumab in 15 patients with metastatic cancer who had progressed on previous ICI-containing treatment. The combination of nadunolimab with pembrolizumab was generally well-tolerated and safe, and treatment resulted in durable disease control for several patients. Serum concentration levels of nadunolimab and pembrolizumab were as expected [16, 31], with no indication of interference in the respective analyses of the two study drugs and no indication of rapid clearance due to anti-drug antibody formation. No safety concerns were identified.

The patient population was heterogeneous both in regard to cancer type and previous treatments, although all study participants had reported disease progression after at least 12 weeks of previous ICI treatment, suggesting acquired resistance to ICI. The prognosis for this group was expected to be poor, and only modest clinical benefit was anticipated. In this light, the observed median OS of 19.7 months in our study appears very promising, albeit the small population, and contrasts with several KEYNOTE studies that reported median survival rates of 8–10.5 months in patients with a range of solid tumors and past treatment exposures [32–34]. Our results suggest that nadunolimab in combination with pembrolizumab may confer a clinically relevant benefit to patients who have previously progressed on ICI-containing treatments. The relatively short median iPFS of 3.4 months, as compared to median OS of 19.7 months, is in line with previous publications of immunotherapy trials where improvements in OS without concomitant improvements in PFS have been noted [35–37]. This may be due to the unique mechanism of action of ICIs and other anti-tumor drugs that act on the cell composition in the TME, rather than by direct cytotoxicity. As previously published, we also observed that a lower tumor burden at baseline correlated with longer survival [38, 39].

The expression of PD-L1 was generally low in the pre-treatment tumor biopsies, suggesting that some tumors may have lost their PD-L1 expression, although our data suggest that PD-L1 expression might be restored over time. The low PD-L1 expression and the acquired resistance to ICI might explain why no correlation between response and the expression level of PD-L1 at baseline was observed in our study, unlike other studies [40–42].

Consistent with previous reports [16–18], we observed IL1RAP, the nadunolimab target, on tumor cells, immune cells, and stroma cells in all three cancer types. No correlation between the IL1RAP level and outcome was observed in this study, in contrast to a previous study in metastatic pancreatic cancer [28, 43], possibly due to the small and heterogeneous patient population. By contrast, immune cell populations in the tumor appeared to influence the clinical benefit achieved by nadunolimab and pembrolizumab treatment. Patients who lived longer had more NK cells in their baseline tumor biopsies, supporting the ADCC mechanism of action of nadunolimab. The smaller baseline tumor size in patients with higher levels of NK cells might also indicate that these tumors are more susceptible to nadunolimab and pembrolizumab treatment. Patients with longer survival and greater tumor shrinkage also appeared to have higher levels of CD8 + T cells at baseline, which are important for the ICI anti-tumorigenic immune response [44]. The observed increase in systemic T cell-related cytokines with treatment suggests a treatment-induced effect on T cell activation. The higher levels of CD163 + immune cells (M2 macrophages) in the patients with longer survival could indicate signs of an immune-suppressed TME, as M2 macrophages are usually associated with immune suppression, increased tumor aggressiveness, early recurrence, and a poor prognosis [45, 46]. This immunosuppression might be counteracted by nadunolimab and pembrolizumab treatment, since blockade of the IL- 1 family potentiates ICI treatment by inhibiting immune suppression by myeloid cells in mice [6, 9, 19, 47]. IL1RAP is highly expressed on myeloid cells and treatment effects were seen with a reduction in circulating monocytes, neutrophils, and eosinophils, while leaving the lymphocyte population intact. This observation supports a role for IL1RAP in regulating myeloid cells that might be involved in immunosuppression. The observations of on-treatment reductions in IL- 6 and lower NLRs in patients with the longest OS are in agreement with this role and with previous reports of these parameters as prognostic markers [48, 49].

In conclusion, the combination of nadunolimab with pembrolizumab was well-tolerated in patients with advanced solid tumors who had received previous treatment with PD- 1/PD-L1 inhibitors. Our study is limited by the small sample size, heterogeneous population, and the lack of a control group. Nonetheless, we observed an apparent survival benefit in the group of patients with high levels of macrophages and NK cells in the TME at baseline and who developed reduced circulating levels of IL- 6 following treatment. These observations may reflect both the IL- 1 inhibition and the ADCC functionality of nadunolimab. The data in our study support further exploration of the combination of nadunolimab and ICIs in advanced cancers. Moreover, from a broader view, these data contribute to our understanding of the importance of TME and its modulation for a successful anti-cancer therapy.

## Supplementary Information

Below is the link to the electronic supplementary material.

Supplementary file1 (DOCX 413 KB)

## Data Availability

The anonymized datasets generated and/or analyzed during the current study are available from the corresponding author on reasonable request.

## Abbreviations

ADCC: Antibody-dependent cellular cytotoxicity
CI: Confidence interval
DLT: Dose or treatment limiting toxicity
HNSCC: Head and neck squamous cell carcinoma
iDCR: Immune disease control rate
IL: Interleukin
IL1RAP: IL-1 receptor accessory protein
iPFS: Immune progression-free survival
NK: Natural killer
NSCLC: Non-small cell lung cancer
PD: Progressive disease
PD-L1: Programmed death ligand 1
PR: Partial response
OS: Overall survival
SD: Stable disease
TEAE: Treatment-emergent adverse event
TME: Tumor microenvironment
TNFα: Tumor-necrosis factor-alpha
