# Supplementary material for: Safety, tolerability, and preliminary efficacy of nadunolimab, an anti-IL- 1 receptor accessory protein monoclonal antibody, in combination with pembrolizumab in patients with solid tumors
Source: Invest New Drugs. 2025 May 1;43(3):609–20. doi: 10.1007/s10637-025-01538-3 (PMC12310844; doi:10.1007/s10637-025-01538-3)
Supplement: Supplementary file 1 — Supplementary file1 (DOCX 413 KB) [file 10637_2025_1538_MOESM1_ESM.docx]

***Investigational New Drugs***

**SUPPLEMENTARY MATERIAL**

**Safety, tolerability, and preliminary efficacy of nadunolimab, an anti-IL-1 receptor accessory protein monoclonal antibody, in combination with pembrolizumab in patients with solid tumors**

Roger B Cohen, Antonio Jimeno, Jennifer Hreno, Lova Sun, Marie Wallén-Öhman, Camilla Rydberg Millrud, Annika Sanfridson, Ignacio Garcia-Ribas

**Corresponding author:** Roger B Cohen, Division of Hematology-Oncology, University of Pennsylvania, Philadelphia, PA. Phone: +215-662-4469 [Roger.Cohen@pennmedicine.upenn.edu](mailto:Roger.Cohen@pennmedicine.upenn.edu).

### Inclusion criteria

1. To be eligible to participate in this study, candidates were to meet the following criteria: Ability to understand and willingness to provide written informed consent before any trial-related activities (trial-related activities are any procedures that would not have been performed during normal management of the subject
2. Age ≥ 18 years
3. Subjects with metastatic or locally advanced, incurable disease (NSCLC [adenocarcinoma, adenosquamous, or squamous], HNSCC, urothelial cancer, or malignant melanoma) who have either exhausted or declined to receive available standard therapy that has demonstrated an improvement in OS.
4. Subjects progressing on previous treatment with a checkpoint inhibitor targeting the PD-1/PD-L1 pathway, alone or in combination with chemotherapy after previously having achieved SD or better and stayed on such therapy for ≥12 weeks.
5. Measurable disease by iRECIST on CT or MRI scan. Imaging tests outside the screening period are valid if performed not more than 2 weeks before consent signature and otherwise fulfill protocol criteria.
6. ECOG performance status ≤1.
7. Primary or metastatic lesion amenable to biopsy and willingness to undergo repeat biopsies, unless a biopsy would not be safe in the opinion of the investigator and after review and agreement by the sponsor and medical monitor (or designee). The lesion accessible for biopsy may not be the only target lesion and should not be located in a previously irradiated field (unless this index lesion has PD ≥20% post radiation). Ideally, the same lesion should be biopsied before treatment and on treatment whenever possible.
8. Clinically adequate bone marrow, hepatic, renal, and coagulation function based on clinical laboratory test values at screening within the following ranges:
   1. Serum creatinine <1.5 × ULN and for part 1 creatinine clearance ≥ 30 mL/minute calculated; for part 2 creatinine clearance ≥ 45 mL/minute. Calculated by the Cockcroft-Gault formula or directly measured with 24-hour urine collection.
   2. Hemoglobin >85 g/L (whole or partial blood [e.g.., red blood cell or platelet] transfusions not allowed 2 weeks before Cycle 1 Day 1)
   3. Absolute neutrophil count >1.5 × 109/L (growth factors like G-CSF are not allowed 2 weeks before Cycle 1 Day 1)
   4. Part 1: Platelets >75 × 109/L; Part 2: Platelets >100 × 109/L. (transfusions not allowed 2 weeks before Cycle 1 Day 1)
   5. Total bilirubin <1.5 × ULN unless due to Gilbert’s syndrome
   6. AST and ALT ≤3 × ULN (for subjects with hepatic metastases <5 × ULN)
9. QT interval corrected with the Fridericia formula (QTcF) ≤ 480 milliseconds at screening.
10. Women of child-bearing potential and men with sexual partners who are women of child-bearing potential and must be willing to adhere to contraceptive requirements from at least 1 month prior to study entry to at least 4 months after the last dose of study treatment.
11. Suitable venous access for safe drug administration and the study-required drug concentration and pharmacodynamic sampling.

**Exclusion Criteria**

Subjects meeting any of the following criteria will be excluded from the study:

1. Subjects with NSCLC tumors harboring aberrations for which FDA-approved targeted therapy is available (known activating EGFR mutations, EGFR T790M mutation, ALK rearrangement, ROS rearrangement, BRAF V600E mutation)., c-met exon 14 mutation) unless approved therapies have been exhausted, are contraindicated, or refused by the subject.
2. Treatment with systemic anticancer treatments, investigational products, or major surgery within 4 weeks before the first dose of study drug or 5 half-lives, whichever is shorter. Subjects should have recovered from previous treatment toxicity to grade 1, baseline (except alopecia and peripheral neuropathy).
3. History of uncontrolled brain metastasis. Subjects with brain metastases are allowed if they are previously treated with surgery, whole-brain radiation, or stereotactic radiosurgery and considered controlled without corticosteroids with ≤10 mg/day of prednisone-equivalent at the time of receiving the first dose of nadunolimab. For asymptomatic subjects, screening brain imaging is not required.
4. Subject has received extended field radiotherapy ≤4 weeks before the start of treatment (≤2 weeks1 week for limited field radiation for palliation), and who has not recovered to grade 1 or better from related side effects of such therapy (except for alopecia).
5. Subjects who, according to the currently approved Keytruda (pembrolizumab) USPI, had an irAE for which permanent discontinuation is mandated (any grade 4 event and grade 3 events of pneumonitis, adrenal insufficiency, hyperthyroidism, hypophysitis, and nephritis). Also, subjects without formal contraindication due to previous irAE are not eligible if the AE has not resolved to grade 1 or better and/or still requires steroids (>10 mg of prednisone equivalent per day) for ongoing management.
6. Active severe infection requiring parenteral antibiotics or subjects currently receiving oral antibiotics as a continuation of a previous course of parenteral antibiotics. Subjects can be enrolled when antibiotic treatment is complete and if there are no signs of residual infection.
7. Evidence of serious uncontrolled medical disorder that, in the opinion of the investigator or medical monitor, makes it unwise for the subject to participate in the study or that might jeopardize compliance with the protocol.
8. Psychiatric illness/social circumstances that would limit compliance with study requirements and substantially increase the risk of AEs or had compromised ability to provide written informed consent.
9. Clinical evidence of an active second invasive malignancy with the exception of stable prostate cancer on watchful waiting.
10. Uncontrolled or significant cardiovascular disease defined as NYHA classification III or IV.
11. History of autoimmune disease requiring systemic immunosuppressive therapy (daily prednisone equivalent doses >10 mg/day).
12. Known hepatitis B virus surface antigen seropositive or detectable hepatitis C infection viral load. Note: Subjects who have positive hepatitis B core antibody or hepatitis B surface antigen antibody can be included but must have an undetectable hepatitis B viral load.
13. Patients positive for human immunodeficiency virus (HIV) are NOT excluded from this study, but HIV-positive patients must meet the following criteria:
    1. have CD4+ T-cell (CD4+) counts ≥350 cells/µL.
    2. have not had an opportunistic infection within the past 12 months. Patients on prophylactic antimicrobials can be included in the trial.
    3. should be on established antiretroviral therapy for at least 4 weeks.
    4. have an HIV viral load less than 400 copies/mL prior to enrolment.
14. Known history of any other relevant congenital or acquired immunodeficiency other than HIV infection.
15. Subjects who receive a live vaccination, etanercept, or other TNF-α inhibitors during or just prior to (within 28 days of first study drug administration) participation in this study.
16. Subjects who have had a hospitalization for bowel obstruction within 12 weeks prior to enrolment.
17. Known bleeding disorder or coagulopathy. Subjects on stable anticoagulant therapy are allowed.
18. Known or suspected allergy to study treatment or related products.
19. Women who are pregnant or breastfeeding or trying to become pregnant.

**Protocol amendment**

The protocol was amended to add part 2 which planned to enroll additional patients with metastatic non-squamous NSCLC to assess the safety of nadunolimab in combination with pembrolizumab, carboplatin and pemetrexed. Only one patient was enrolled under part 2 prior to discontinuation of the study by the sponsor due to commercial reasons. This patient experienced a best response of SD and had no TESAEs or related grade ≥3 TEAEs. No safety concerns were identified.

**Dose selection**

The 5 mg/kg starting dose of nadunolimab was half of the maximum dose tested and shown to be safe during phase 1 monotherapy escalation [18]. This dose was considered biologically active based on non-clinical data and when used in combination with chemotherapy in patients with pancreatic cancer [18, 36]. Dose escalation was not planned. The study included a safety lead-in phase in six participants who received 5 mg/kg nadunolimab with pembrolizumab before expanding to a total of 15 patients.

**Serum concentrations of nadunolimab and pembrolizumab**

Patient serum samples were collected before, and 1 hour after dosing on designated days. Concentrations of nadunolimab were measured at a central laboratory using a chemiluminescence assay with a lower limit of quantification (LLOQ) of 2000 ng/mL. Pembrolizumab was measured by a standard enzyme-linked immunosorbent assay by a central laboratory with an LLOQ of 30 ng/mL.

**Supplemental Table 1** Demographic and baseline disease characteristics (Safety population)

| **Variable** | | **Total**  **N=15** | |
| --- | --- | --- | --- |
|  | |  | |
| Age (years) mean (SD) | | 64.2 (7.7) | |
| Sex, n (%) | |  | |
| Male | | 11 (73%) | |
| Female | | 4 (27%) | |
| Race, n (%) | |  | |
| White | | 14 (93%) | |
| Asian | | 1 (7%) | |
| Stage IV at study entry | | 15 (100%) | |
| ECOG at screening | |  | |
| 0 | | 1 (7%) |  |
| 1 | | 14 (93%) |  |
| Tumor type | |  | |
| HNSCC | | 9 (60%) | |
| Melanoma | | 1 (7%) | |
| NSCLC – adenocarcinoma | | 4 (26%) | |
| NSCLC – squamous | | 1 (7%) | |
| Previous therapy | |  | |
| Tumor burden*, median [range] | | 50 [16, 125] | |
| Radiotherapy | | 9 (60%) | |
| Surgery | | 7 (47%) | |
| Systemic therapy | | 15 (100%) | |
| PD-L1 expression at baseline | |  | |
| <1% | | 4 (27%) | |
| 1-49% | | 7 (47%) | |
| ≥50% | | 0 | |
| Not available | | 4 (27%) | |

ECOG Cooperative Oncology Group score; HNSCC, head and neck squamous cell carcinoma; mOS, median overall survival of 19.7 months; NSCLC, non-small cell lung cancer; OS, overall survival; PD-L1, programmed cell death-ligand 1; SD, standard deviation

*sum of diameters

**Supplemental Table 2** Summary of patients with treatment-emergent adverse events reported by at least two patients, regardless of causality (safety population)

|  | **Total**  **N=15** | |
| --- | --- | --- |
| Any TEAE | 15 | |
| Grade 3/4 | 7 | |
| Grade 5 | 1 | |
| Grade 3/4 TEAEs related to treatment | 2 | |
| Any TESAE | 6 | |
| DLT | 1 | |
| TEAEs leading to treatment discontinuation | 1 | |
|  | **Total**  **N=15** | |
| **Preferred Term** | **Grade 3/4** | **All Grades** |
| Fatigue | 1 | 8 |
| Pruritus |  | 6 |
| Hypotension |  | 4 |
| Arthralgia |  | 4 |
| Dyspnoea | 1 | 4 |
| Diarrhoea |  | 4 |
| Weight decreased |  | 3 |
| Dysphagia | 2 | 3 |
| Rash |  | 3 |
| Muscle spasms |  | 3 |
| Dizziness |  | 3 |
| Urinary retention |  | 2 |
| Muscular weakness |  | 2 |
| COVID-19 | 1 | 2 |
| Oropharyngeal pain |  | 2 |
| Blood creatinine increased |  | 2 |
| Vomiting |  | 2 |
| Pneumonia | 1 | 2 |
| Haematuria |  | 2 |
| Atrial fibrillation |  | 2 |
| Decreased appetite |  | 2 |
| Productive cough |  | 2 |
| Cough |  | 2 |

Abbreviations: DLT, dose or treatment limiting toxicity; TEAE, treatment-emergent adverse event; TESAE, treatment-emergent serious adverse event

Graded using the NCI Common Terminology Criteria for Adverse Events v 5.0.

**Supplemental Table 3** All grade 3, 4 or 5 treatment-emergent adverse events (TEAEs) and serious TEAEs regardless of causality by frequency, by worst grade

|  | **Total**  **N=15** |
| --- | --- |
| **Total number of grade ≥3 TEAEs ​** | 21​ |
| Number of subjects with at least 1 grade ≥3 TEAE | 7 |
| Dysphagia​ | 2 |
| Respiratory failure​ | 1 |
| Pneumonitis​ | 1 ​ |
| Hypoxia​ | 1 ​ |
| Dyspnea​ | 1 ​ |
| Inguinal hernia​ | 1 ​ |
| Covid-19​ | 1 ​ |
| Sepsis​ | 1 ​ |
| Pneumonia​ | 1 ​ |
| *Pneumocystis jirovecii* infection​ | 1* ​ |
| Device-related infection​ | 1 ​ |
| Febrile neutropenia​ | 1 ​ |
| Neutropenia​ | 1 ​ |
| Fatigue​ | 1 ​ |
| Failure to thrive​ | 1 ​ |
| Hypercalcemia​ | 1 ​ |
|  |  |
| **Total number of TESAEs** ​ | 13 |
| Number of subjects with at least 1 TESAE | 6 |
| Dysphagia | 2 |
| Gastrointestinal hemorrhage | 1 |
| Pneumonia | 1 |
| COVID-19 | 1 |
| *P. jirovecii* infection | 1 |
| Sepsis | 1 |
| Device-related infection | 1 |
| Respiratory failure | 1 |
| Dyspnea | 1 |
| Febrile neutropenia | 1 |
| Failure to thrive | 1 |

Percentages are based on the number (N) of included subjects. n: Number of subjects with an event. When a subject experienced more than 1 event in different preferred terms or within the same preferred terms, all incidences are counted.

Graded using the NCI Common Terminology Criteria for Adverse Events v 5.0.

*Grade 5

**Supplemental Table 4** Demographic and baseline characteristics of patients with OS below or above median

| **Variable** | **Patients with OS > mOS**  **N = 7** | **Patients with OS < mOS**  **N=8** |
| --- | --- | --- |
|  |  |  |
| Age (years) mean (SD) | 65.3 (9.3) | 63.3 (5.1) |
| Sex, n (%) |  |  |
| Male | 5 (71%) | 6 (75%) |
| Female | 2(29%) | 2 (25%) |
| Race, n (%) |  |  |
| White | 7 (100%) | 7 (87%) |
| Asian | 0 | 1 (13%) |
| Stage IV at study entry | 7 (100%) | 8 (100%) |
| ECOG at screening |  |  |
| 0 | 1 (14%) | 0 |
| 1 | 6 (86%) | 8 (100%) |
| Tumor type |  |  |
| HNSCC | 4 (57%) | 5 (56%) |
| Melanoma | 1 (14%) | 0 |
| NSCLC – adenocarcinoma | 2 (29%) | 2 (25%) |
| NSCLC – squamous | 0 | 1 (13%) |
| Previous therapy |  |  |
| Tumor burden*, median [range] | 37.9 [16, 50] | 80.3 [46, 125] |
| Radiotherapy | 3 (43%) | 6 (75%) |
| Surgery | 3 (43%) | 4 (50%) |
| Systemic therapy | 7 (100%) | 8 (100%) |
| PD-L1 expression at baseline |  |  |
| <1% | 1 (14%) | 3 (38%) |
| 1-49% | 3 (43%) | 4 (50%) |
| ≥50% | 0 | 0 |
| Not available | 3 (43%) | 1 (13%) |

ECOG Cooperative Oncology Group score; HNSCC, head and neck squamous cell carcinoma; mOS, median overall survival of 19.7 months; NSCLC, non-small cell lung cancer; OS, overall survival; PD-L1, programmed cell death-ligand 1; SD, standard deviation

*sum of diameters

**Supplemental Fig. 1** Prior treatments and length of treatment in patients participating in CIRIFOUR

**Supplemental Fig. 2** Overall survival and iPFS by tumor type in patients treated with nadunolimab with pembrolizumab


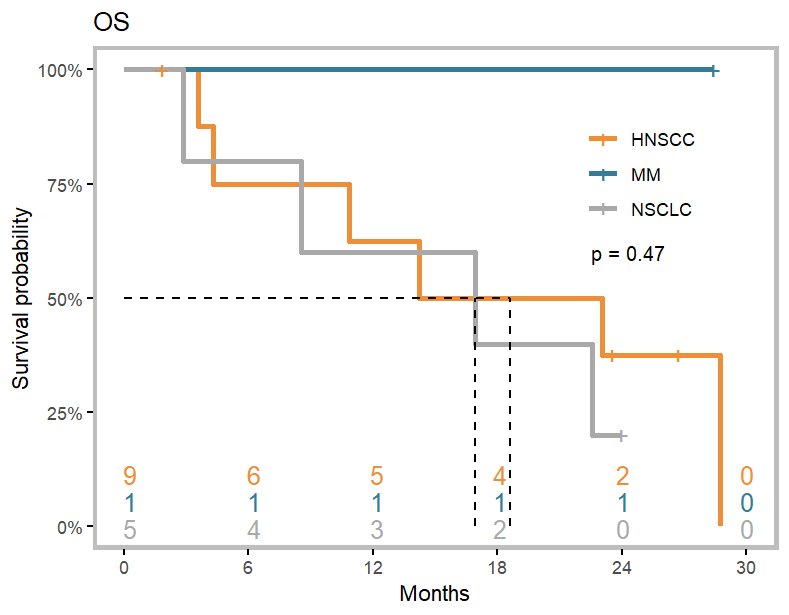

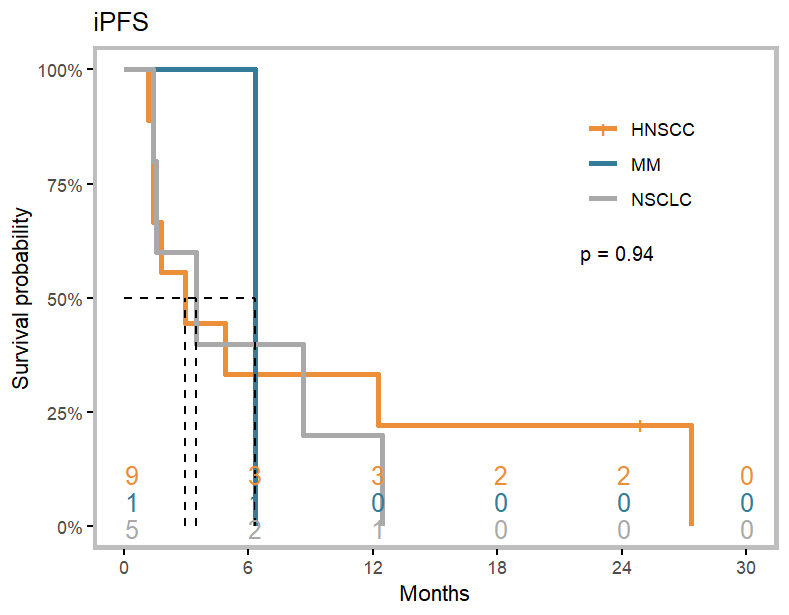


HNSCC, head and neck small cell cancer; MM, malignant melanoma; NSCLC, non-small cell lung cancer;

OS overall survival; iPFS, immune progression-free survival

**Supplemental Fig. 3** Nadunolimab and pembrolizumab serum concentrations over 10 treatment cycles.


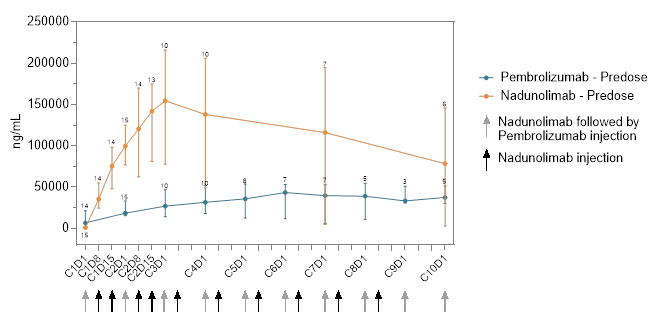


Median serum concentration profiles from blood samples taken prior to each dose of nadunolimab (orange) and pembrolizumab (blue) for the first 10 cycles, with 95% confidence intervals. Patients received weekly dosing of nadunolimab in cycles 1 and 2, nadunolimab twice per cycle in cycles 3-8, and once per cycle in cycles 9-10.

Arrows below indicate dosing schedule of nadunolimab (black arrows) and nadunolimab + pembrolizumab (grey arrows). Numbers above error bars show the number of patients at each time point.

Note that baseline values for pembrolizumab may have been influenced by residual levels from previous treatments, with 9 of 15 patients treated with pembrolizumab within 100 days of enrollment in the current trial.

**Supplemental Fig 4.** Target expression of IL1RAP and PD-L1 in tumor biopsies at baseline and on treatment

**
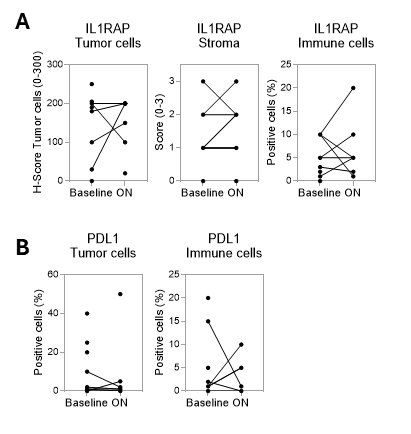
**

Patient biopsies were collected at screening (n=12) and on treatment (cycle 2 day 8; n=8) and stained for IL1RAP and PD-L1 by immunohistochemistry. **(A)** IL1RAP expression was evaluated on tumor cells, stroma, and immune cells in baseline and on treatment (ON) biopsies. **(B)** PD-L1 expression was evaluated on tumor cells and immune cells in baseline and on treatment (ON) biopsies.

IL1RAP, interleukin 1 receptor accessory protein; PD-L1, programmed cell death ligand 1

**Supplemental Fig. 5** Levels of CD8+ T cells, CD163+ macrophages and NK cells in the tumor microenvironment at baseline and on-treatment

**
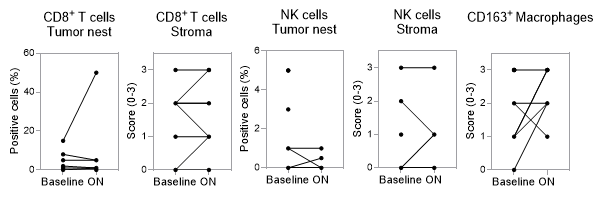
**

Biopsies were collected at screening (Baseline; n=12) and on-treatment (ON; cycle 2 day 8; n=8) and stained for the expression of CD8, NKp46 or CD163 using immunohistochemistry to evaluate the presence of CD8+ T cells, NK cells and M2 macrophages in the tumor. Percentage of CD8+ immune cells and NKp46+ immune cells were evaluated in tumor nest and stromal area separately, while the CD163 staining was scored as 0-3 in the whole biopsy.

All screening biopsies were evaluated for CD8+ and CD163+ immune cells, whereas 11 were evaluated for the levels of NKp46+ immune cells due to exhaustion of one NSCLC sample.

NK, natural killer; ON, on treatment

**Supplemental Fig. 6** Nadunolimab induces cytokine secretion in whole blood from healthy individuals

**
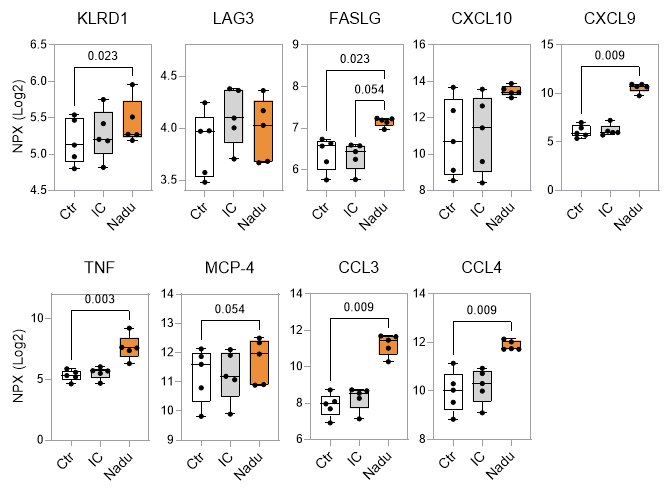
**

Whole blood from five healthy donors was treated with nadunolimab (30 µg/ml), isotype control (30 µg/ml) or left untreated for 18 h, supernatants harvested and analyzed for cytokine levels using Olink Target 96 Immuno-Oncology panel. NPX, Normalized Protein expression.

**Supplemental Fig. 7** Correlations of biopsy expression of PDL1 and IL1RAP with overall survival


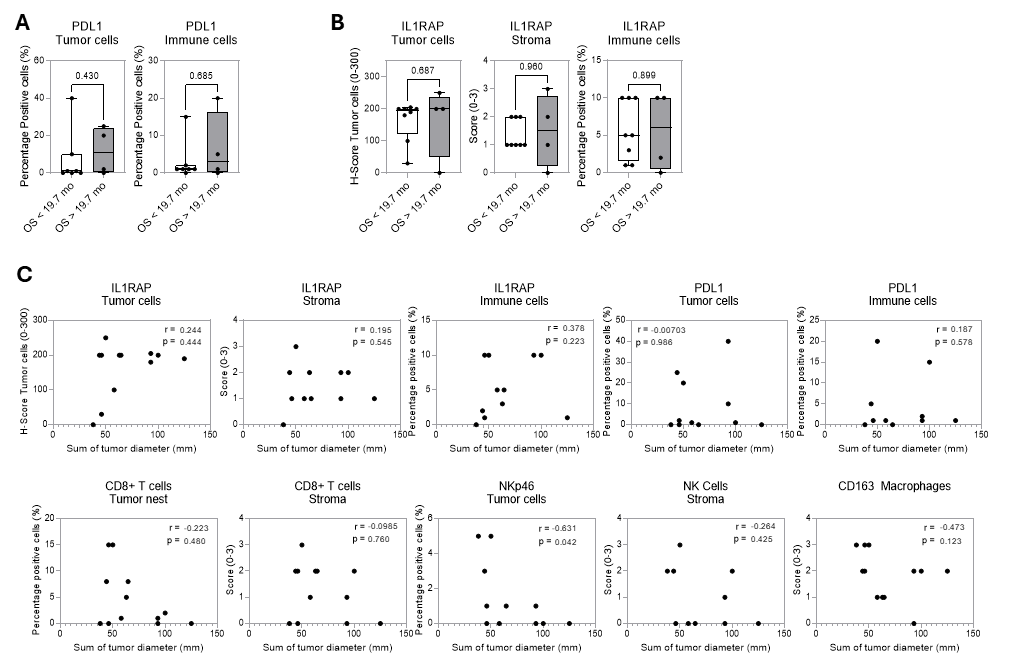


Patients were divided on median OS (19.7 months) into those with longer OS than 19.7 months (OS >19.7 months) and those with shorter OS than 19.7 months (OS <19.7 months) and analyzed for correlation with biopsy expression of IL1RAP and PDL1. **(A)** PD-L1 expression on tumor cells and immune cells and **(B)** IL1RAP expression on tumor cells, stroma or immune cells at baseline was determined in the two groups. **(C)** Correlation between baseline sum of diameter and the different tumor biopsy markers.
